# Does a consumer training work? a follow-up survey of the PartecipaSalute training programs

**DOI:** 10.1186/1478-4505-10-27

**Published:** 2012-09-01

**Authors:** Paola Mosconi, Roberto Satolli, Cinzia Colombo, Walter Villani

**Affiliations:** 1 Laboratory of Medical Research and Consumer Involvement, Mario Negri Institute for Pharmacological Research, Via La Masa 19, Milan, 20156, Italy; 2 Scientific publishing company Zadig, Via Ampére 59, Milan, 20131, Italy; 3 Laboratory of Medical Research and Consumer Involvement, Mario Negri, Institute for Pharmacological Research, Via La Masa 19, Milan, 20156, Italy

**Keywords:** Healthcare, Training program, Consumer, Empowerment

## Abstract

**Background:**

When properly trained through training programs on epidemiology, clinical research and healthcare policy, members of patients’/consumers’ organizations could be helpful for a patient-oriented healthcare system. Since 2006 the not for profit project PartecipaSalute has organized periodic editions of a training program for representatives of citizens’/patients’ organizations. After five editions of this training program, a survey of the long-term satisfaction and the impact on activities has been carried out.

**Methods:**

A 17-questions follow-up questionnaire has been developed. The sample comprised 99 people who had taken part in at least one program edition.

**Results:**

The overall response rate was 89% (89 responders/99 participants). About 98% of participants expressed general satisfaction with the training program and with the knowledge gained. Medical and informative topics were rated better than technical ones for their usefulness (96% versus 86%). The results of the survey showed a strong impact of the training course on single participants, while a weak outcome on the activities of the organizations was reported.

**Conclusions:**

The training program was positively rated, and improvements in personal knowledge were reported. Less impact was reported on organizations’ activities. Participants showed a remarkable willingness to get more involved in healthcare decisions, and to boost their knowledge of health and research issues. The results show the importance of follow-up to understand the real value of training program and to better organize future programs.

## Background

Putting the consumer or patient at the center of healthcare systems is fundamental in the planning and evaluation of health services [1-5]. Consumers or patients and their representatives, when properly informed and trained, can help to support an effective need and care based healthcare system [1-5]. Consequently, empowerment on healthcare and clinical research issues is vital to make sure consumers or patient centrality is really considered as an important resource for the debate and not only a purely formal presence [6,7]. Consumers’ and patients’ organizations therefore have a bridging role between social and medical assistance and healthcare planning and management [8,9].

Consumers/patients - in particular their representatives - must acquire the skills of “experts”, collaborating with each other and integrating their different viewpoints, knowledge and experience [9-11]. In fact, the expertise they have is a unique and precious resource, completely different from health professionals’ and health managers’ skills. However, to make full use of them, consumers, patients and organization members need to grasp some basic scientific and medical concepts and critical appraisal skills. Institutions collaborating with consumers’/patients’ organizations and research groups are in fact organizing more training programs and debates, on the methods of epidemiological and clinical research and on healthcare policy [12-14].

Some important experiences are the Leadership, Education and Advocacy Development-LEAD Project [15], Europa Donna training for breast cancer patients organizations [16], or Rare Diseases Europe-Eurordis for rare disease patients organizations [17] or recently a peer education training program [18]. However, it is hard to find data on the impact of training programs in term of gains and advantages, evaluated as the internal or external activities of the organizations involved.

In Italy a large number of people are involved in voluntary work (3.3 million according to the latest report of the Italy’s Central Institute of Statistics) and more than 25% of volunteers are connected with health associations [19]. Over the years, the “agenda” and the priorities of associations have shifted from being conservative, where members mainly provide assistance, to lay projects, where consumers’ rights and participation in health are debated.

Since 2006 PartecipaSalute - a not-for-profit research project designed to foster a strategic alliance among healthcare professionals, patients, and their organizations, developing activities with different levels of involvement [20] – has organized training programs for representatives of citizens’ and patients’ organizations [21]. The aim of the training course is to increase the critical thinking skills on important issues and controversies in health debate, and to encourage the autonomy of representatives of citizens’ and patients’ organizations to be engaged in different forum or committees, and effectively influence health decisions.

After five editions, we now consider it essential for planning future editions to make a survey on long-term evaluation of the impact of these activities on participants and their organizations.

## Materials and methods

The aim of the present study is to evaluate the follow-up in terms of impact on participants’ activities and satisfaction.

Three programs were organized at Mario Negri Institute for Pharmacological Research in Milan, one in Tuscany [22] and one in Emilia-Romagna. The training program is modular, with one topic for each module. Each module starts with working groups, common discussion in plenary session, formal lessons and ends with a debate. We invite each participant to take an active part, starting from his/her own experience. Researchers, health professionals, representatives of patients’ organizations, lay people on ethics committees, and medical journalists serve as speakers and tutors. The entire training program is free; materials such as slides, scientific literature and an *ad hoc* manual published by PartecipaSalute [23] is distributed to participants. These materials can also be downloaded from the project’s website (www.partecipasalute.it).

The program is promoted through websites, mailing lists or personal contacts. Applicants were selected on the basis of specific criteria, such as geographical distribution of the centers, health-generic or disease-specific organizations, self-evaluated healthcare-related knowledge, and availability to follow all the modules of the program. People with a professional healthcare background were excluded because they could have influenced the discussion, in particular during working group activities.

During each edition of the training program, participants rated their satisfaction with tutors and topics using a self-administered forms, on a Likert scale; effects on knowledge were also evaluated with a before/after test [22].

**Figure 1:**
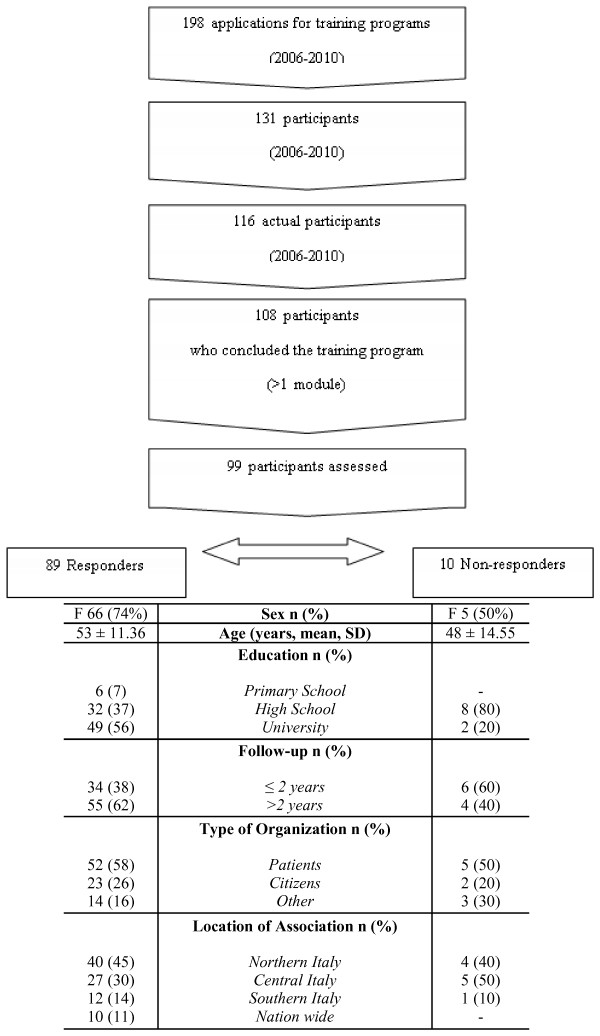
Study flowchart and participants’ main details.

For the years 2006–2010 we collected 198 candidates; for this follow-up study, the sample is made up of all 131 people who had participated and completed at least one edition of the training program (Figure 1). Fifteen were excluded as redundant and 8 did not complete the programs. This left 108 participants who were all contacted. Nine were then excluded because they no longer worked in the same organization or because they had health problems, leaving a sample of 99 participants.

To assess the satisfaction and the impact for all five editions of the training program, a follow-up questionnaire has been developed, starting from a critical revision of participants’ comments collected during each edition of the training course. The first version has been revised by two PartecipaSalute project researchers (CC and RS). The second version has been sent for a face validity phase to three reviewers selected from the GRAL group (Gruppo Rappresentanti Associazioni e Laici) – a group of patients and consumers supporting PartecipaSalute project [21]. At the end of this phase, all the comments have been collected and discussed, and the final version has been defined. The 17 questions - 14 dichotomic yes/no and 3 multiple-choice questions - focused on three main areas: a) general satisfaction with the training program, b) impact of the training, c) impact on the organizations represented by participants (see Appendix  1).

The survey was conducted from February 2011 to April 2011. The sample comprised 99 people who had taken part in at least one program edition, all were initially contacted by e-mail. Several reminders were sent: one by e-mail (10 days after the first), two by telephone (after 20 days and 40 days) and one by fax and telephone text message (55 days after the first e-mail).

Completed forms were collected and all data were transferred into a dedicated electronic database for the analysis. For descriptive analysis, absolute frequencies were used for all categorical variables; central trend and dispersion measurement (mean, standard deviation) were used for quantitative continuous variables. The χ^2^ test for association was used to compare the patterns of answers with socio-demographic variables. Data were analyzed using the statistical package SAS 9.1 (Statistical Analysis System, SAS Institute Inc., Cary – NC, USA).

## Results

Of the 99 participants, 89 responded to the questionnaires (responders), with a final response rate of 89% (Figure 1). The follow-up ranged from one to five years and it covered more than two years after the training program for 62% of responders. Almost three-quarter of responders were women (74%) and more than half had a high level of education; nearly 60% were members of patients’ organizations. All participants represented a voluntary association (see list in the Acknowledgments section). Thirty one of the 75 (41%) responders were leaders of their own organization.

Table 1 shows all the results of the questionnaires. Almost all participants (87/88, i.e. 99%) reported they were been satisfied with the program, and a similar percentage was satisfied with the knowledge gained and most considered their health knowledge had improved. A very large majority expressed interest in participating in new editions.

**Table 1:** Satisfaction and impact among participants

**General satisfaction with the training program**	N* (%)
Improving health knowledge (Yes)	87/88 (99)
Re-using the material distributed (Yes)	83/89 (93)
General satisfaction	
Very poor	-
Poor	1/89 (1)
Good	32/89 (36)
Very good	56/89 (63)
Satisfaction with health knowledge	
Very poor	-
Poor	2/89 (2)
Good	36/89 (41)
Very good	51/89 (57)
Interest in a new future edition of the training program (Yes)	78/89 (88)
**Impact of the training on participants ( Yes)**	
Taking new roles in the organization	20/88 (23)
Representing the organization at congresses, meetings and seminars	55/89 (62)
Promoting changes in the organization or its direction in accordance with issues covered in the training program	52/88 (59)
Distributing all or part of the training program material to other members of the association	54/87 (62)
Changes in relationships with clinicians in the association’ Scientific Committee	23/85 (27)
Changes in the relationship with institutions, research centers and scientific society	30/86 (35)
**Impact on the organization (Yes)**	
Reporting after the training all activities to other members of the association	78/88 (89)
Promoting activities or events in accordance with the training	46/84 (55)
Developing a network between different associations	46/84 (55)
Frequently visiting the websites suggested during the training program	36/84 (43)

* Responders/number of participants, some data are missing.

Table 2 shows participants’ satisfaction with each modules. “*Uncertainties in medicine*” and “ *Information and education in healthcare*” were the most appreciated, both considered useful by 96% of participants. The least appreciated module was “ *Internet in medicine and research*” which was rated as useful by two third of responders, with a useful : not useful ratio less than 2:1.

**Table 2:** Usefulness of training program topics for the organization’s daily work

**Training program topics**	Useful N* (%)	**Ratio**
		**(Useful:Not useful)**
Uncertainties in medicine	81/84 (96)	27 : 1
Information and formation in healthcare	83/88 (94)	17 : 1
Conflicts of interest	75/83 (90)	9 : 1
ABC of the research	73/85 (86)	6 : 1
Ethics Committee	68/85 (80)	4 : 1
Internet in medicine and research	53/83 (64)	2 : 1

* Responders/number of participants, some data are missing.

Regarding the impact on participants (Table 1), only 23% (20/88) of responders changed their role in the voluntary association they represented (“I have a more active role: after the course I can inform and teach other members better”, “I shared the training course experience with other members and now I’m monitor for the internal education”). However after training, 62% (55/89) represented their organization at congresses, meetings and seminars, and 59% proposed some changes in the organization’s policies, in accordance with issues treated in the programs (“I asked my organization to act with more transparency in case of conflicts of interest”, “I ask my association to invest more in education and training”). Moreover, 27% (23/85) and 35% (30/86) of the participants considered their relationships had changed, respectively with clinicians and with institutions. Participants older than 55 years changed their role in the organization significantly (<0.05) more frequently than younger ones. No real differences were found for other socio-demographic variables.

Regarding the impact on the organizations 89% (78/88) of responders reported their experience to other organization members, and 55% (46/84) stated their organizations had promoted some activities or events in line with topics dealt with during the programs. The same percentage (55%) reported they had set up networks with different organizations, but less than the half (43%, i.e. 36/84) reported other members of their organizations visited websites suggested during the training.

## Discussion

This study reports the results of a follow-up survey carried out on a sample of representatives of citizens’/patients’ organizations participating to a training program on healthcare and clinical research issues. *Ad hoc* training programs for patients’ and consumers’ organizations are to be considered as milestones for an appropriate empowerment process [24]. This method of involving patients and consumers is considered essential to support participation in debates on the healthcare and on the priorities of research [6]. Although many countries experience training programs - particularly UK, Canada, USA, and Australia where trainings are considered integral part of healthcare policies [15,18] – in our knowledge no data are available on the impact and satisfaction with participants, especially when organized groups (such as associations of citizens and patients) are directly involved. The findings of this study, although related to Italy, underline the importance and relevance of training programs for patients’ and consumers’ organizations, and point out important issues to be considered in organizing training programs. First, the evaluation of the training impact is essential, both on the organizations (cascade effect), and on the single participants. Secondly, in order to better evaluate the impact, the evaluation has to be defined from the very beginning, in the organization of the program, and training sessions on how to disseminate the training program in each patients organization have to be scheduled. Finally, each participant should indicate an activity she/he will organize to spread in the organization the topics covered by the training course, and the promoters should follow-up it.

This survey on the satisfaction and impact of the PartecipaSalute training programs shows a high level of general satisfaction, indicating that consumers and patients appreciated these empowerment process. The response rate to the survey is also very high. Despite these encouraging general results, not all the topics in the program were rated equally regarding their usefulness for the daily work of organizations. More technical issues, as “*ABC of the research*”, “ *Ethics Committee*” and “ *Internet in medicine and research*”, received relatively lower ratings, probably reflecting the difficulties of teaching and understanding these topics. Results suggest that members of organizations prefer topics bridging medicine and social science, indicating their interest in acquiring more interpretative skills than deeper discussions on scientific methods and results. In addition, as data reported, our experience suggests that internet and its potential are not yet considered so valuable as expected among representatives of organizations.

All responders were interested in participating in future editions, demonstrating the true essence of empowerment: not just a passive involvement into a process aimed to gain more technical knowledge, but an incessant process aimed to increase ability and awareness with the final goal to become more autonomous from healthcare professionals, and to better participate in informed decisions.

Due to this cascade effect on other volunteers and other close organizations, this consideration get more important when representatives of organizations are directly involved in the empowerment process. Our experience shows that participants reported improvement in personal knowledge on treated topics, whereas the impact on activities and “feelings” of organizations seemed weaker. Though a large percentage of responders represented and supported their organizations in public meetings, and individually promoted changes in their organizations in line with the program’s topics, a small percentage reported changes in relationships with clinicians and institutions. Similarly, although participants said they had reported the training activities to other members, only about the half said their organizations promoted activities and events or developed a network among associations. This is important for groups and institutions involved in empowerment of members of patients’ organizations. For this reason the training programs must have to cover not only the single participant but also organization, involving them much more actively.

Considering the results obtained from this survey, PartecipaSalute will include in future editions: a section aimed to discuss how to implement the course contents, in terms of conveying information to other members of organization or change routine practices. Moreover, we decided to prepare a slide information kit with the essential key messages of the training course to facilitate the cascade effect. Finally, we are discussing the possibility to ask to each participant the development of an initiative to do in partnership with the organization, helping in the design and development of the idea.

The training program of PartecipaSalute is supported by an independent not-for-profit organization and there is currently no similar initiative at institutional level in Italy. To organize successful empowerment programs tailored for patients and consumers, appropriate resources, personnel and time should be located and considered as critical elements [24]. In Anglo-Saxons countries, institutions, as Involve project [25] or Food and Drug Administration [26], invest and dedicate considerable amount of resources in this area of interest. Even pharmaceutical companies founding training activities for patients’ organizations [27]. In Italy, one of the main obstacles to develop and organize new training initiatives is the modest interest of government institutions, - and as a consequence scant funding for these projects. To date, only two editions of the training program have been partially supported by public regional grants (Regione Toscana, Edition IV) or local or provincial grants (Regione Emilia-Romagna, Rimini, Edition V). The fourth edition, organized in collaboration with the Regione Toscana, had two modules centered on clinical risk issues, aiming to set up a group of empowered consumers to be involved in the clinical risk monitoring. Now, two years after the program, a local network of empowered associations has been established, and joint work has organized such as in audits, the communication of adverse events, and sharing assessment tools [28].

This study has some limits, first this experience is limited to the Italian setting and the survey is done on a small sample of 89 people who followed the training course. Second, after each PartecipaSalute training course was not requested a formal monitoring of the activities or discussions produced after the training course and this could be a bias for the responses to the questionnaire. Third, details about some topics, like changes in relationship with clinicians and institutions, were not collected. Finally, in the literature there are no other similar data to compare the results obtained.

## Conclusions

These results, though we are fully aware that are limited and specific to a PartecipaSalute training course, indicate that patients and citizens are remarkably willing to get much more involved in healthcare decisions and to improve their knowledge on health and research issues. We would underline the importance of the evaluation of impact of training programs. In our opinion a better understanding of the cascade effect is necessary to strength the effort to generate empowerment. So we think that our study could be useful to enforce the methodological background in planning future training course, even at international level.

## Competing interests

The authors declare that they have no competing interests.

## Authors’ contributions

PM and WV made substantial contributions to the conception, design, analysis and interpretation of results and write-up of the manuscript. CC and RS contributed to the interpretation of results in addition to write-up of the final version of the paper. All authors read and approved the final manuscript.

## Supplementary Material

Additional file1 Partecipasalute survey. Click here for file
