# Supplementary material for: Does a consumer training work? a follow-up survey of the PartecipaSalute training programs
Source: Health Res Policy Syst. 2012 Sep 1;10:27. doi: 10.1186/1478-4505-10-27 (PMC3464673; doi:10.1186/1478-4505-10-27)
Supplement: Additional file1 — Partecipasalute survey. [file 1478-4505-10-27-S1.doc]

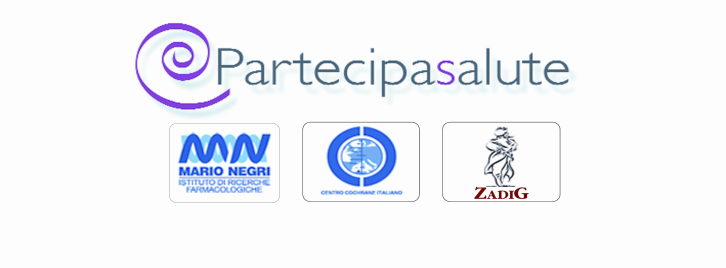
**PARTECIPASALUTE SURVEY**

**Responder …………………………………………………………………**

•**1.** Do you believe that the PartecipaSalute training course has increased your knowledge on health topics?

Yes No

•**2.** Did you use the material distributed during the PartecipaSalute training course? Yes No

If Yes, please specify how *(More answers are possible)*

- Used for personal interest
- Used for search specific information
- Used for presentations and/or write articles
- Other, please specify ……………………………………………………………………………………...

•**3.** After the experience in the PartecipaSalute training course, did your role within your organization change?

Yes No

If Yes, please specify how your role is changed …………………………………………………………

………………………………………………………………………………………………………………

•**4.** After the experience in the PartecipaSalute training course, did you accept new assignments in working groups, courses, seminars, committees as member of your organization?

Yes No

•**5.** Are you generally satisfied with the PartecipaSalute training course?

Very poor Poor Good Very good

•**6.** With regard to knowledge acquired, are you satisfied with the course?

Very poor Poor Good Very good

•**7.** As member of the organization, and considering all the issues covered in the PartecipaSalute training course, which ones have been the most useful in your activity?

Very useful Useful Not much useful Not useful

- ABC of research clinic

- Uncertainties in medicine

- Conflicts of interest

- information and formation in health care

- Ethics committee

- Internet and research in medicine

•**8.** If possible, would you participate in a new edition of the PartecipaSalute training course?

Yes No

•**9.** After this experience, and according with the issues addressed in the PartecipaSalute training course, have you promoted initiative for introducing changes within your organization?

Yes No

If Yes, please specify ………………………………………………………………………………………

•**10.** After the experience in the course, did your organization asked you to report, or you have independently reported to your association, about the issues of the PartecipaSalute training course?

Yes No

If Yes, through which methods? *(More answers are possible)*

- Organized meetings - Has written a report / report
- He spoke generically - Wrote articles

**If No, Please go to the question 12**

•**11.** If you reported about the issues PartecipaSalute training course, did the organization, or the members, show interest in the issues addressed?

Yes No

•**12.** After the experience in the PartecipaSalute training course, did you distribute or circulate among the members of your organization, the materials distributed/discussed during the course?

Yes No

•**13.** After the experience in the course, did your organization promote initiatives in line with the PartecipaSalute training course ?

Yes No

If Yes, please specify:

- Promoted /participated in randomized clinical trials (RTC)
- Wrote /distributed information materials new or revised
- Wrote /published a document concerning the conflict of interest
- Started a collaboration with institutions
- Started a collaboration with some scientific society
- Organized meetings /conferences /courses
- Published articles for the organization's Web site
- Created links to the website shown during the training course
- Other ....................................................................................................................................................................

If Yes, briefly describe the experiences:

………………………………………………………………………………………………………………………………….

………………………………………………………………………………………………………………………………….

•**14.** After your participation to the PartecipaSalute training course, did the relationship with doctors of the Scientific Committee of your organization change?

Yes No I don’t know

If Yes, how?

- They has improved
- They got worse

•**15.** After the experience in the PartecipaSalute training course, did the relationships with the institutions, scientific societies or centers nursing reference changed?

Yes No I don’t know

If Yes, how?

- They are improved
- They are worse

•**16.** After the experience in the PartecipaSalute training course, did your organization decide to "network", i.e. developed strengthening the cooperation with other organization?

Yes No I don’t know

If Yes, please specify ……………………………………………………………………………………….

………………………………………………………………………………………………………………

•**17.** After the experience in the PartecipaSalute training course, did you, or your organization, regularly visit the website PartecipaSalute and / or other websites recommended during the course?

Yes No

If Yes, please specify ………………………………………………………………………………….........

………………………………………………………………………………………………………………

**Thanks for your kind collaboration**
